# PerC Manipulates Metabolism and Surface Antigens in Enteropathogenic *Escherichia coli*

**DOI:** 10.3389/fcimb.2017.00032

**Published:** 2017-02-07

**Authors:** Jay L. Mellies, Amy Platenkamp, Jossef Osborn, Lily Ben-Avi

**Affiliations:** ^1^Biology Department, Reed CollegePortland, OR, USA; ^2^Molecular Microbiology and Immunology, Oregon Health and Science UniversityPortland, OR, USA

**Keywords:** Pathogenic *E. coli*, EPEC, PerC, *fim* switch, type I fimbriae, nitrate reduction, niche adaptation, virulence

## Abstract

Enteropathogenic *Escherichia coli* is an important cause of profuse, watery diarrhea in infants living in developing regions of the world. Typical strains of EPEC (tEPEC) possess a virulence plasmid, while related clinical isolates that lack the pEAF plasmid are termed atypical EPEC (aEPEC). tEPEC and aEPEC tend to cause acute vs. more chronic type infections, respectively. The pEAF plasmid encodes an attachment factor as well as a regulatory operon, *perABC*. PerC, a poorly understood regulator, was previously shown to regulate expression of the type III secretion system through Ler. Here we elucidate the regulon of PerC using RNA sequencing analysis to better our understanding of the role of the pEAF in tEPEC infection. We demonstrate that PerC controls anaerobic metabolism by increasing expression of genes necessary for nitrate reduction. A tEPEC strain overexpressing PerC exhibited a growth advantage compared to a strain lacking this regulator, when grown anaerobically in the presence of nitrate, conditions mimicking the human intestine. We show that PerC strongly down-regulates type I fimbriae expression by manipulating *fim* phase variation. The quantities of a number of non-coding RNA molecules were altered by PerC. In sum, this protein controls niche adaptation, and could help to explain the function of the PerC homologs (Pch), many of which are encoded within prophages in related, Gram-negative pathogens.

## Introduction

The Pch (PerC homolog) family of proteins is found in multiple, pathogenic members of the *Enterobacteriaciae*. These include different *Escherichia* pathotypes, *Shigellae, Salmonella*, and *Klebsiella* species. The majority, if not all of the genes encoding this family of proteins are found on horizontally transferred elements: Plasmids and within prophage. Little is known about the structure or function of these relatively small proteins, ~10 kDa, except that they control transcriptional activity of established virulence genes in two *E. coli* pathotypes.

The *perABC* operon was originally described as a regulator of the *eae* locus of enteropathogenic *E. coli* (EPEC) (Gómez-Duarte and Kaper, 1995). Subsequently, it was shown that PerC increases expression of *eae*, encoding intimin, indirectly through activation of the *LEE1* operon, encoding the master regulator Ler (Mellies et al., 1999; Bustamante et al., 2001, 2011). The LEE, or locus of enterocyte effacement, encodes a type III secretion system necessary for virulence, the protein intimin, and is necessary for tight attachment in the formation of attaching and effacing lesions on the intestine epithelium (Elliott et al., 2000). EPEC net secretory diarrhea occurs by three main mechanisms: Destruction of the microvilli leading to maladsorption, alteration of host cell signaling events leading to ion secretion and water loss, and loosening of the tight junctions (Clarke et al., 2003; Kaper et al., 2004; Santos and Finlay, 2015).

Typical EPEC (tEPEC) contain the pEAF plasmid, while there are also clinically significant strains that lack this virulence plasmid, and are termed atypical EPEC (aEPEC). In tEPEC, the 89-amino acid PerC protein is encoded on the 97-kb pEAF virulence plasmid. This plasmid, which also encodes the bundle-forming pilus, or BFP, contains an IncF1b origin of replication and is estimated to be present in 2–5 copies per cell (Gibbs et al., 1993; Tobe et al., 1999; Iguchi et al., 2009). Subsequently to the identification of PerC in EPEC, five *pch* genes were identified in *E. coli* O157:H7, a serotype causing hemorrhagic colitis, along with the serious complication known as hemolytic uremic syndrome, or HUS (Frankel et al., 1998; Nataro and Kaper, 1998; Ogura et al., 2006). These *pch* genes homologs are all found within cryptic prophage in this hemorrhagic pathotype, or EHEC bacterium. Porter et al. demonstrated that (PerC1) PchA, PchB, and PchC of EHEC were interchangeable with PerC of EPEC in their ability to activate transcription of *LEE1* in both pathotypes, illustrating a conservation of function (Porter et al., 2005). These investigators found that the combined expression of prophage-encoded PchA, PchB, and PchC was roughly equivalent to that of the plasmid-encoded PerC of EPEC, implicating gene dosage as being important for downstream regulatory effects. These studies introduced a family of transcriptional regulators, but their overall function in the *Enterobacteriaciae* family remained unclear.

Plasmids are thought to confer bacterial adaptations to local niches (Eberhard, 1989). Plasmid-encoded regulators often control other plasmid-encoded genes, but also can control chromosomally encoded regulatory elements and genes that enable adaptation to locally restricted environments. As an example, *Rhodococcus equi* is an intracellular pathogen of macrophage that contains a conjugative virulence plasmid encoding a 21-kb pathogenicity island necessary for growth within macrophage (Coulson et al., 2015). The plasmid-encoded VirRS regulatory proteins control ~18% of chromosomally-encoded genes, integrating control of nutrient transport, energy production, and metabolism. In EPEC, PerC integrates regulatory control of the chromosomally-located LEE pathogenicity island through direct modulation of the Ler (Mellies et al., 1999; Bustamante et al., 2001, 2011; Iyoda and Watanabe, 2004; Porter et al., 2005; Adler et al., 2014). Though there exists precedent for plasmid-encoded regulatory genes controlling virulence traits, such as fimbrial antigens, and central metabolism, the potential role for PerC in these functions remains unexplored.

To gain better insight into a function that the Pchs perform in *Enterobacteriaciae* pathogens, we used RNAseq analysis to determine the set of PerC-regulated genes in EPEC. We provide evidence that PerC increases expression of genes necessary for nitrate reduction, and decreased *fim* expression by biasing the genetic switch *fimS* toward the OFF position. As nitrate is an important terminal electron acceptor in the distal small intestine, the site of EPEC infection, and control of extra-cellular adhesin biosynthesis is linked to pathogenesis, we assert that the plasmid-encoded PerC of EPEC is involved in niche adaptation via these mechanisms.

## Materials and methods

### Bacterial strains and growth

The bacterial strains and plasmids used in this study are listed in Table 1. Unless otherwise indicated, overnight liquid cultures were grown in lysogeny broth (LB) at 37°C with 225 rpm shaking and, in the case of the strains containing plasmids pMPM-T3 and pTEPPerC1, 15 μg/ml tetracycline. Strains were also grown on LB agar plates at 37°C with and without appropriate antibiotic selection. Low-glucose Dulbecco's modified Eagle's medium (DMEM) was prepared from DMEM-D2902 (Sigma-Aldrich, St. Louis) containing 0.1% D-glucose and supplemented with 25 mM HEPES, pH 7.4 and 44 mM NaHCO_3_. For growth in DMEM, strains were first propagated overnight in LB with appropriate antibiotics, and then sub-cultured as indicated.

**Table 1:** **Strains and plasmids used in this study**.

**Strain or plasmid**	**Genotype or description**	**Source**
**STRAINS**		
***Escherichia coli***		
E2348/69	WT EPEC, *Str^*R*^*	Levine, 1987
JPEP22	E2348/69 Δ*perC*::*Kan^*R*^, Str^*R*^*	Bustamante et al., 2011
DH5α	*Str^*R*^*	Lab stock
ORN172	Δ*fimBEACDFGH, Kan^*R*^*	Bäumler et al., 1996
MC4100		Lab stock
KH4100	MC4100 *ara^+^*	Mellies et al., 2008
***Fusobacterium nucleatum***		
VPI 4355	Obligate anaerobe	ATCC 25586
**PLASMIDS**		
pMPM-T3	p15A derivative, *Tet^*R*^*	Mayer, 1995
pTEPPerC1	pMPM-T3 derivative, *perC, Tet^*R*^*	Bustamante et al., 2011
pRK2013	Helper plasmid, RK2 replicon, *Kan^*R*^*	Figurski and Helinski, 1979
pJLM164	*LEE1::lacZ*	Mellies et al., 1999

### Genetic procedures

The *perC*-containing pTEPPerC1 plasmid was constructed previously (Bustamante et al., 2011), and is a derivative of pMPM-T3 (Mayer, 1995), a pBluescript vector. Though pTEPPerC1 contains a *lac* promoter, *perC* expression occurs constitutively in the absence of inducer (Bustamante et al., 2011). The pMPM-T3 and the derivative pTEPPerC1 plasmids in DH5α were transferred into the Δ*perC* mutant strain JPEP22 via bacterial conjugation with a separate DH5α containing the helper plasmid pRK2013 necessary for mobilization of non-self-transmissible plasmids (Figurski and Helinski, 1979). The three strains were incubated together at 37°C overnight on LB and then plated on LB plates supplemented with tetracycline (15 μg/ml) and kanamycin (50 μg/ml) to select for successful conjugation.

### Anaerobic growth assay

For anaerobic growth in the presence of nitrate, after overnight growth in LB, cultures were diluted to an optical density at 600 nm (OD_600_) of ~1 and subcultured 1:50 in Tryptic Soy Broth (TSB; CellGro, Manassas, VA, 61-412-RO) containing 0.5% (w/v) mucin type III partially purified from porcine stomach (Sigma-Aldrich, St. Louis, MO), and Oxyrase Enzyme System for Broth Media (Oxyrase, Mansfield, OH) as per the manufacturer's instructions. Sodium nitrate (40 mM; NaNO_3_) was added where indicated. Strains were inoculated in biological duplicate and technical triplicate for all four conditions and cultured at 37°C to mid exponential growth phase at which PerC is strongly expressed (OD_600_ 0.3–0.5) (Bustamante et al., 2011). The obligate anaerobic bacterium *Fusobacterium nucleatum* subspecies nucleatum strain VPI 4355 (ATCC 25586, American Type Culture Collection, Manassas, VA) was incubated in the presence and absence of Oxyrase to determine efficacy of oxygen-scavenging. *F. nucleatum* grew in the presence of Oxyrase, thus media with Oxyrase was determined sufficiently anaerobic.

### Competition assay

For competition assays, after overnight incubation in LB with antibiotic selection, strains were adjusted to the same OD_600_ of 0.1. Each culture (1 ml) was used to co-inoculate one 10-ml culture of low-glucose DMEM. Cultures were statically incubated at 37°C with 5% CO_2_, and dilutions were plated on LB agar and on LB agar supplemented with kanamycin (50 μg/ml) at 0 and 4 h post-inoculation to determine growth in colony-forming units (CFU) per ml. The assay was also performed in DMEM containing 40 mM NaNO_3_ and statically grown with 5% ambient CO_2_. This protocol was adapted from Portal-Celhay and Blaser (2012).

### RNA sequencing and whole-transcriptome analysis

Flasks containing low-glucose DMEM were inoculated 1:100 with an overnight culture of WT EPEC or the Δ*perC* deletion strain and incubated with 225 rpm shaking at 37°C to mid-exponential phase (OD_600_ 0.3–0.5). Incubation and RNA harvesting of three biological replicates were carried out for all samples simultaneously and under RNase-free conditions to minimize variability in gene expression between each sample (Hansen et al., 2011). Bacterial concentrations were equalized to the same density (by OD600). Samples were then diluted 1:1 in RNA Protect (Qiagen, Carlsbad, CA) to inhibit RNase activity and then centrifuged (5000 × g, 10 min). Pellets were resuspended in TE/lysozyme (10 mg/ml lysozyme, 0.5% SDS, pH 8.0) with added proteinase K (1.5 mg/ml). RNA was isolated using Qiagen's RNeasy kit according to manufacturer instructions with slight modification. Before centrifugation, β-mercaptoethanol was added to RNeasy kit buffer RLT (10% v/v). Additionally, during the wash step RNase-free DNase (Qiagen, Carlsbad, CA) was diluted in RNeasy kit buffer RDD (310 Kunitz units/mL), added to the purification column, and incubated for 15 min at room temperature (RT). After isolation, spectrophotometric NanoDrop (NanoDrop 1000 v3.8.1; Thermo Fisher) curves were obtained for each RNA sample and verified for purity, as defined by absorbance ratios at 260/280 nm and 260/230 nm. RNA samples were sent to Oregon State University's Center for Genome Research and Biocomputing. RNA integrity (RIN) measurements were taken using an Agilent Bioanalyzer, resulting in RIN scores of 10, out of a possible 10, for each sample. Ribosomal RNA was depleted using the RiboZero rRNA removal kit (Life Technologies, Eugene, OR), and resulting mRNA was reverse transcribed to cDNA libraries via SuperScript III First Strand reverse transcription kit (Invitrogen, Carlsbad, CA) as per the manufacturer's instructions. The cDNA libraries were multiplexed to distinguish replicates from one another, barcoded for sequencing, and then amplified with random hexamers for 15 PCR cycles. Transcripts were sequenced for 50 bases in single-end fashion within one lane of an Illumina HiSeq 2000 flow cell. This yielded roughly 30 million reads per sample, which is more than sufficient to identify differential gene expression in the EPEC transcriptome (Haas et al., 2013; Mcclure et al., 2013). Ninety-five percent of the reads were mapped to the EPEC strain E2348/69 genome, and the reads were trimmed using the software Trimmomatic (Bolger et al., 2014). Only those with scores >30 were used in the analysis and no mismatches were allowed.

### Reverse transcription quantitative PCR

Overnight cultures of the 4 experimental strains in biological duplicate were diluted to an OD_600_ of 1 and sub-cultured 1:50 in low-glucose DMEM. Strains Δ*perC* (p*perC*) and Δ*perC* (pVector) were grown with added 15 μg/ml tetracycline in both cases. DMEM cultures were statically grown to an OD_600_ of 0.3–0.5 at 37°C with 5% CO_2_.

Each sample (2.5 × 10^9^ cells, ~4 ml) was diluted 1:1 in RNAprotect Reagent (Qiagen, Carlsbad, CA). Samples were prepped for isolation by vortexing for 5 s and incubating at RT for 5 min. Cultures were centrifuged at 12,000 rpm at 4°C for ~30 min. The supernatant was decanted and samples were resuspended in 200 μl of 0.5 mg/ml lysozyme (Sigma-Aldrich, St. Louis, MO) with 0.2 μg/ml proteinase K (Sigma-Aldrich) in TE pH 8.0 (Thermo Fisher Scientific). Samples were incubated at RT for 10 min on a shaking incubator and vortexed every 2 min during incubation. Lysis buffer (200 μl) was added. RNA was automatically isolated using the Maxwell® 16 (Promega, Madison, WI) and the Maxwell® 16 LEV simplyRNA Tissue Kit per the manufacturer's protocol. RNA was re-suspended in 50 μl of RNase-free TE. Genomic DNA was degraded by DNaseI (New England Biolabs, Ipswich, MA) digestion per the manufacturer's protocol. No reverse-transcriptase controls proved adequately low levels of genomic DNA (data not shown). Concentration and quality of RNA was determined by NanoDrop, and RNA across samples was diluted to the same concentration. RNA was reverse-transcribed into a cDNA library using random hexamer primers and the SuperScript III First Strand reverse transcription kit (Invitrogen, Carlsbad, CA) per the manufacturer's instructions.

Quantitative PCR was performed on a Roche LightCycler 480 and analyzed in Roche 1.5.1.62 SP2 software using the ΔΔC_t_ method to determine relative transcript abundance of target genes, in biological replicates technically triplicated (the *napC* experiment was technically duplicated), against the reference 16S ribosomal *rrsB* gene. Efficiency (100 ± 10%) was determined for each primer pair (Table 2). For one reaction, cDNA (2 μl) was reacted with 7.5 μl 2X ImmoMix (Bioline, London, UK), 2 μl forward primer (2 μM; Invitrogen), 2 μl reverse primer (2 μM; Invitrogen), 1.5 μl 5X SYBR Green (Invitrogen S7563), and 2.5 μl water. The cycling parameters were as follows: (1) 50°C for 2 min, (2) 95°C for 10 min, (3) 95°C for 15 s, (4) 55°C for 30 s, (5) 72°C for 30 s with a single fluorescence acquisition, (6) GO TO step 3 for 39 more cycles, and (7) 72°C for 10 min. A melting curve was performed after each run to assure product homogeneity. The melting curve parameters were as follows: (1) 95°C for 5 s, (2) 65°C for 1 min, increasing by 0.11°C increments per second from 60 to 95°C while continuously analyzing the fluorescence at a rate of 5 acquisitions/°C, and (3) product was then cooled at 40°C for 10 s.

**Table 2:** **Oligonucleotides used in this study**.

**Primer**	**Target**	**Sequence (5′-3′)**
**RT-qPCR**		
FimAJO516Fwd	*fimA*	TTAGGACAGGTTCGTACCGC
FimAJO516Rev	*fimA*	TCGCCGTACCTAAAAAGGCA
NapCJO516Fwd	*napC*	GGTTTGCCCATCTTTCACCG
NapCJO516Rev	*napC*	AGGACAACAACTCGCGGAG
NarKJO516Fwd	*narK*	CGCTGTTGCGGTGAACTTAC
NarKJO516Rev	*narK*	CGACGACCACCGAAGATAGG
rrsB_RT_5end_fwd	*rrsB*	AGTTATCCCCCTCCATCAGG Xue et al., 2015
rrsB_RT_3end_rev	*rrsB*	TGCAAGTCGAACGGTAACAG Xue et al., 2015
***fimS*** **assay**		
OL4 fimS Forward	*fimS*	CCGTAACGCAGACTCATCCTC Corcoran and Dorman, 2009
OL20 fimS Reverse	*fimS*	GAGTTTTAATTTTCATGCTGCTTTCC Corcoran and Dorman, 2009

### *fimS* phase variation assay

A PCR-based assay described previously was used to determine the effect of PerC on genomic *fim* phase variation. Overnight cultures of WT EPEC, Δ*perC*, Δ*perC* (p*perC*), and Δ*perC* (pVector), were diluted 1:100 in LB or DMEM and incubated at 37°C with 225 rpm shaking to mid-exponential phase (OD_600_ 0.4–0.5), when PerC is preferentially expressed (Bustamante et al., 2011). Bacterial concentrations were equalized to an OD_600_ of 0.5, samples were boiled for 5 min, and *fimS* DNA was amplified by endpoint PCR using primers described previously (Corcoran and Dorman, 2009) (Table 2). Products were separated on 1% agarose in TAE. The PCR products were then incubated with the restriction enzyme BstUI (New England Biolabs, Ipswich, MA) at 60°C overnight. The products of this digestion were separated on 1% agarose, and the resulting band patterns were used to determine the phase of *fimS*.

### Data analysis

For the whole-transcriptome analysis, sequencing was verified using FASTQC (version 0.11.2) (Andrews, 2010) to confirm base-call quality in each indexed file. Indices corresponding to the same sample were merged, and then uploaded to a cloud-based variant of Galaxy named RNA23 Rocket (Giardine et al., 2005; Blankenberg et al., 2010; Goecks et al., 2010; Warren et al., 2015). Using Bowtie2 (version 2.0.2) (Langmead and Salzberg, 2012), sequenced reads were mapped to an EPEC O127:H6 reference genome (EMBL/GenBank accession codes FM180568, FM180569, and FM180570). Mapped reads were quality checked with SAMstat (version 1.08), and transcripts were assembled in Cufflinks (version 2.0.2) (Trapnell et al., 2010; Lassmann et al., 2011). Differential gene expression (DGE) was then performed on fragments per kilobase mapped (FPKM) values in Cufflinks' Cuffdiff package (version 0.0.7). DGE analysis used a false-discovery rate (FDR) of 0.10. Subsequent data visualizations were performed in R (version 3.1.2) using the cummeRbund package (version 3.0) (Goff et al., 2013; R Development Core Team, 2015).

β-galactosidase activity was analyzed by a Student's *t*-test in JMP (version 11) (SAS Institute Inc)^1^ with a *p*-value < 0.05 considered significant.

RT-qPCR data were normalized to the 16S ribosomal gene *rrsB* and analyzed by the ΔΔC_q_-method. The mean log_2_ fold-change was analyzed in a student's *t*-test in JMP (version 11) (SAS Institute Inc) in which values were compared to the no-change value of expression. A *p*-value of < 0.05 was considered significant.

Growth curves were analyzed by linear regression in Prism 7.0 (GraphPad Software)^2^ to determine growth rate (slope of linear fit) discrepancies between conditions in exponential phase. A *p*-value of < 0.05 was considered significant. Competition assays were subjected to Student's *t*-test in JMP (version 11) (SAS Institute Inc) where a *p*-value of < 0.05 was considered significant.

### Data sharing

The RNA sequencing data, Series record GSE91001, was submitted to the Gene Expression Omnibus (GEO) repository supported by NCBI on Wednesday, December 7, 2016, and is scheduled to be released to the public on Feb 07, 2017. Raw FASTQ files and processed bedGraphs of each sample along with a bedGraph file of the averaged sample data from which conclusions regarding expression values were drawn were submitted.

## Results

### Whole-transcriptome analysis

Because PerC of EPEC and the PchABC proteins of EHEC are functionally interchangeable in their ability to activate LEE gene expression (Iyoda and Watanabe, 2004; Porter et al., 2005), and the EPEC allele is located on a virulence plasmid, we used whole-transcriptome analysis to identify a comprehensive set of genes regulated by PerC, ultimately to gain insight into how the Pch proteins regulate virulence. We monitored transcripts in a Δ*perC* mutant strain compared to the WT coisogenic EPEC parent when grown to early exponential phase in DMEM. Given the role of other plasmid-encoded genes in local niche adaptation (Eberhard, 1989), we predicted that PerC would control the expression of genes, in addition to *ler*, that would allow the bacterium to successfully colonize the human intestine.

Differential gene expression (DGE) analysis showed that 157 genes (3.7% of those chromosomally-encoded) were statistically significantly regulated using a false-discovery rate (FDR) of 10% (*q* ≤ 0.10). This corrected for the 5073 unpaired *t*-tests Cuffdiff performed, which compared average fragments per kilobase mapped (FPKM) for each gene in the WT and deletion mutant conditions.

PerC induced the expression of genes involved in anaerobic respiration in the WT condition compared to the coisogenic Δ*perC* mutant (Table 3). This was especially evident in the increased expression of nitrate and nitrite reductases. The genes *napC, napH*, and *napA*, encoding components of a periplasmic nitrate reductase, were increased ~2-fold (*q* = 0.00, 0.00, and 0.04, respectively). As the product of nitrate reduction, nitrite, is toxic at high levels, we expected to observe differential expression of genes encoding both nitrite reductases and transport proteins. Predictably, the *nrfAG* complex that reduces nitrite to ammonia in the periplasm and the *nirBD* transcripts, performing the same task in the cytoplasm, were increased in expression up to 6- and 2-fold, respectively relative to the Δ*perC* strain (Table 3). The *narK* nitrate/nitrite anti-porter, that brings nitrate into the cytoplasm, was also increased in expression 1.51-fold in the WT vs. the Δ*perC* mutant strain. Taken together, these data suggested that PerC enhanced anaerobic respiration using nitrate as a terminal electron acceptor.

**Table 3:** **Whole transcriptome RNA-sequencing indicates that PerC increases transcription of nitrate reductases.^**a**^**.

**Gene ID**	**Name**	**Description**	**Fold change^b^**	**log_2_(fold change)**	**WT FPKM**	**JPEP22 FPKM**	***Q*-value**
E2348C_0385	*glnK*	Nitrogen assimilation regulatory protein for GlnL GlnE and AmtB	2.09	1.07	31.49	15.03	0.02
E2348C_2346	*napC*	Nitrate reductase cytochrome c-type periplasmic	2.31	1.21	169.12	73.06	0.00
E2348C_2348	*napH*	Essential for electron transfer from ubiquinol to periplasmic nitrate reductase (NapAB)	2.23	1.16	249.98	111.88	0.00
E2348C_2350	*napA*	Nitrate reductase catalytic subunit	1.97	0.98	351.80	178.60	0.04
E2348C_1347	*narK*	Nitrate/nitrate transporter	1.51	0.60	125.72	83.18	0.12
E2348C_3615	*nirB*	Nitrite reductase large subunit NAD(P)H-binding	2.60	1.38	536.17	206.05	0.00
E2348C_3617	*nirC*	Nitrite transporter	3.19	1.68	92.61	28.99	0.00
E2348C_4393	*nrfA*	Nitrite reductase formate-dependent cytochrome	4.81	2.27	71.03	14.77	0.00
E2348C_4394	*nrfB*	Nitrite reductase pentaheme subunit	4.39	2.13	21.93	5.00	0.07
E2348C_4395	*nrfC*	Formate-dependent nitrite reductase NrfC 4Fe4S subunit	6.42	2.68	43.02	6.70	0.00
E2348C_4396	*nrfD*	Formate-dependent nitrite reductase NrfD membrane subunit	5.81	2.54	34.06	5.86	0.00
E2348C_4397	*nrfE*	Heme lyase for insertion of heme into c552 subunit	3.81	1.93	8.99	2.36	0.00
E2348C_4501	*nsrR*	Rrf2 family nitric oxide-sensitive transcriptional repressor	1.60	0.68	243.86	152.61	0.07
E2348C_4563	*nrdG*	Anaerobic ribonucleoside-triphosphate reductase activating protein	1.73	0.79	74.36	42.91	0.03
E2348C_4564	*nrdD*	Anaerobic ribonucleoside triphosphate reductase	1.59	0.67	39.74	25.02	0.07

a *Three biological replicates of mRNA were extracted from WT EPEC strain E2348/69 and coisogenic strain JPEP22ΔperC*.

b *Defined as WT divided by ΔperC fragments per kilobase mapped (FPKM)*.

Expression of plasmid-encoded *perA* (–0.6 fold change, n.s.) and *perB* (–2.3 fold change, *q* = 0.00) transcripts were reduced in the WT strain relative to the Δ*perC* mutant (Table S1). PerA has been shown to activate transcription of the *bfp* operon (Bustamante et al., 1998). Indeed, in our analysis several of the genes of the *bfp* operon were significantly reduced in the WT vs. the Δ*perC* mutant, consistent with down-regulation by PerA (Table S1). This suggested a role for PerC in the suppression of auto-activation of the *perABC* operon.

Repression of the *fim*-encoded type I pilus by PerC was the strongest regulation observed by DGE analysis (Table 4). Thus, the Δ*perC* mutants had ~100-fold (*q* < 0.01) increased transcript levels in the major type I pilin subunit encoded by *fimA* compared to the WT strain. The Fim adhesin adheres to host cells via its FimH subunit, which binds mannose-binding receptors on the enterocyte's surface (Krogfelt et al., 1990). The gene *fimH* was decreased in expression ~10-fold when comparing the WT to the Δ*perC* mutant strain. These data suggested that PerC manipulates the expression of EPEC type I pili on the surface of the bacterium.

**Table 4:** **Whole transcriptome RNA-sequencing indicates that PerC decreases transcription of the ***fim*** operon genes.^**a**^**.

**Gene ID**	**Name**	**Description**	**Fold change^b^**	**log_2_(fold change)**	**WT FPKM**	**JPEP22 FPKM**	***Q*-value**
E2348C_4619	*fimB*	Tyrosine recombinase/inversion of on/off regulator of fimA	0.51	–0.98	55.22	109.11	<0.01
E2348C_4620	*fimE*	Tyrosine recombinase/inversion of on/off regulator of fimA	0.19	–2.41	10.19	54.34	<0.01
E2348C_4621	*fimA*	Major type 1 subunit fimbrin (pilin)	<0.01	–8.10	28.31	7744.93	<0.01
E2348C_4622	*fimI*	Fimbrial protein involved in type 1 pilus biosynthesis	0.01	–7.54	1.25	231.58	<0.01
E2348C_4623	*fimC*	Chaperone, periplasmic	0.01	–6.67	1.86	188.99	<0.01
E2348C_4624	*fimD*	Outer membrane usher protein, type 1 fimbrial synthesis	0.02	–6.02	3.31	215.29	<0.01
E2348C_4625	*fimF*	Minor component of type 1 fimbriae	0.05	–4.42	9.80	209.96	<0.01
E2348C_4626	*fimG*	Minor component of type 1 fimbriae	0.10	–3.38	2.69	28.02	<0.01
E2348C_4627	*fimH*	Minor component of type 1 fimbriae	0.10	–3.36	6.10	62.75	<0.01

a *Three biological replicates of mRNA were extracted from WT EPEC strain E2348/69 and coisogenic strain JPEP22ΔperC*.

b *Defined as WT divided by ΔperC fragments per kilobase mapped (FPKM)*.

Lastly, we observed altered expression of a number of Hfq-dependent sRNAs (Table S1). These included *isrA, dsrA, rygC, rygD, rnpB, rhyB*, and *csrC*. While *rhy*B has been implicated in iron homeostasis in EPEC (Adler et al., 2014), it is unclear how the other sRNAs contribute to niche adaptation.

### Reverse transcription quantitative PCR

In order to confirm initial observations made by RNAseq analysis, we performed reverse-transcription quantitative PCR (RT-qPCR). For genetic complementation, the pTEPPerC1 plasmid encoding *perC* and the pMPM-T3 empty vector control were conjugated into the Δ*perC* strain JPEP22 (the resulting strains were termed Δ*perC* (p*perC*) and Δ*perC* (pVector), respectively (Table 1). As a control, we demonstrated that the pTEPPerC1 induced expression of the *LEE1-lacZ* fusion in a K-12-derived strain—the β-galactosidase assay demonstrated that pTEPPerC1 produced 1400 Miller units of activity, while the empty-vector pMPM-T3 produced 140 Miller units, thus β-galactosidase activity was greater in the strain containing pTEPPerC1 than that containing pMPM-T3 (*p* = 0.0006) (Figure S1).

**Figure 1:**
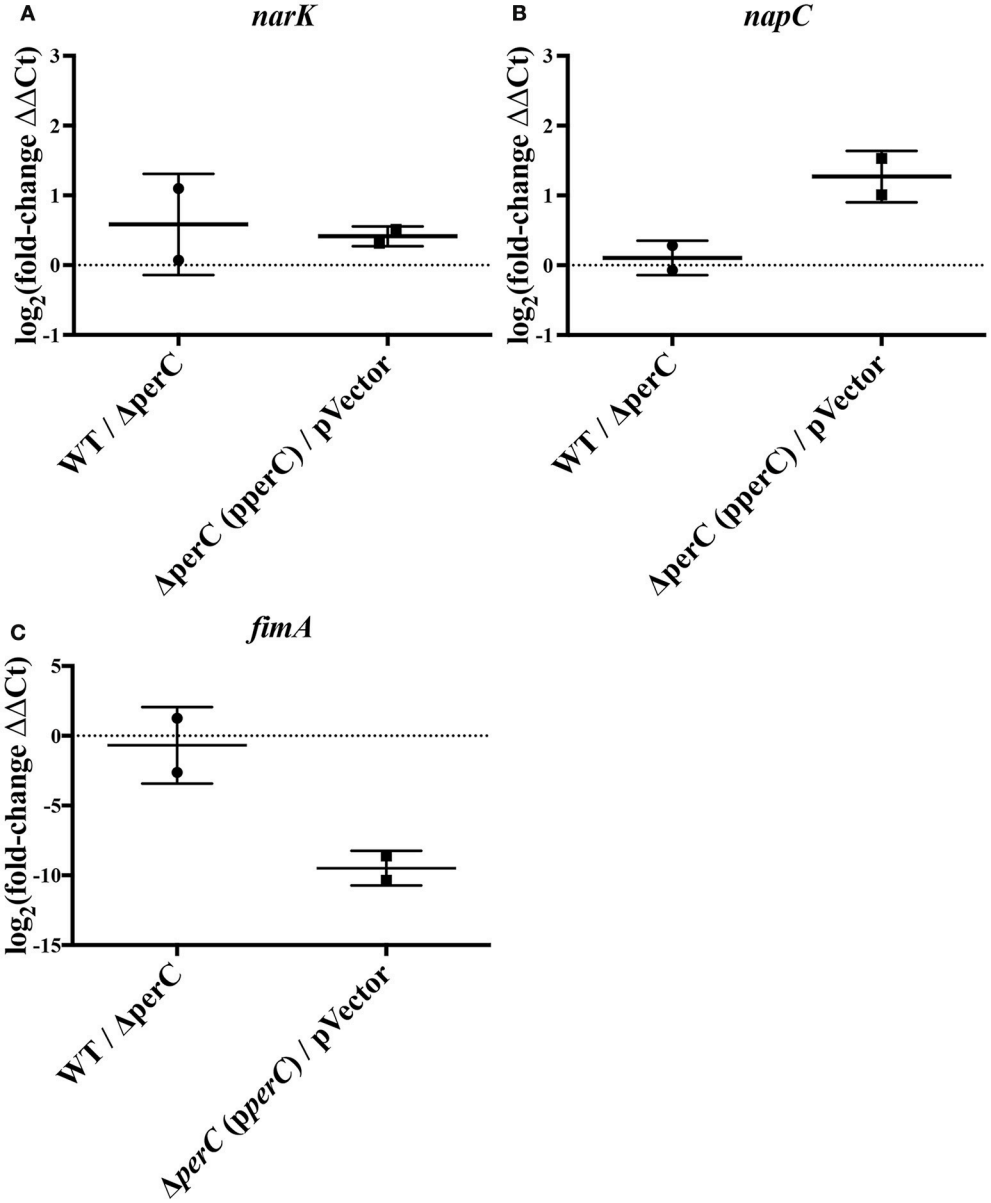
**RT-qPCR indicates level of expression of ***narK, napC***, and ***fimA*** in WT compared to Δ***perC*** mutant and complement compared to empty-vector EPEC strains [log_**2**_(fold change) ± SEM]**. Expression levels of *narK* **(A)**, *napC* **(B)**, and *fimA* **(C)** were normalized to 16S ribosomal reference gene *rrsB* and the fold change was calculated via the ΔΔC_q_-method.

We observed an increase in expression of the nitrate/nitrite anti-porter *narK* and nitrate reductase component *napC* in strains expressing the *perC* gene, consistent with the RNAseq data. By RT-qPCR, there was a significantly greater amount of *narK* transcripts present in the complement strain compared to the empty-vector strain (0.41 ± 0.12 log_2_-fold increase; *p* = 0.0007) as well as in the WT strain compared to the Δ*perC* mutant (0.58 ± 0.33 log_2_-fold increase; *p* = 0.0193) (Figure 1). While the increased expression for *napC* was more modest (0.11 ± 0.23, n.s.) for the WT compared to the Δ*perC* mutant than determined by RNAseq (Figure 1), expression levels for the complement and vector strains were more similar to the RNAseq levels and were statistically significant: 1.3 ± 0.43 log_2_-fold increase (*p* = 0.0233) (Figure 1).

Relative expression of *fimA* was likewise confirmed. RT-qPCR of the type 1 pili subunit indicated an insignificant –0.68 ± 0.98 log_2_-fold decrease of *fimA* expression in the WT strain compared to the Δ*perC* mutant (Figure 1), a somewhat modest change in expression across strains. However, expression in the complement and empty-vector strains proved more similar to data collected by RNAseq: There was a statistically significant –9.5 ± 0.66 log_2_-fold change (*p* = 0.005) (Figure 1). Confirmation of the increased expression of nitrate respiratory genes *narK* and *napC*, along with the decreased expression of *fimA* provided greater confidence in the observed RNAseq data.

### Anaerobic respiration in the presence of nitrate

Based on the data that indicated PerC increased expression of genes necessary for nitrate and nitrite reduction, we predicted that EPEC strains with a functional *perC* gene would exhibit a growth advantage compared to those without it when respiring anaerobically. We conducted our analysis by monitoring growth of strains cultured shaking in LB, statically cultured in low-glucose DMEM, mimicking tissue culture conditions, and then anaerobically in the presence of mucin to model the human intestine (Winter et al., 2013).

**Figure 2:**
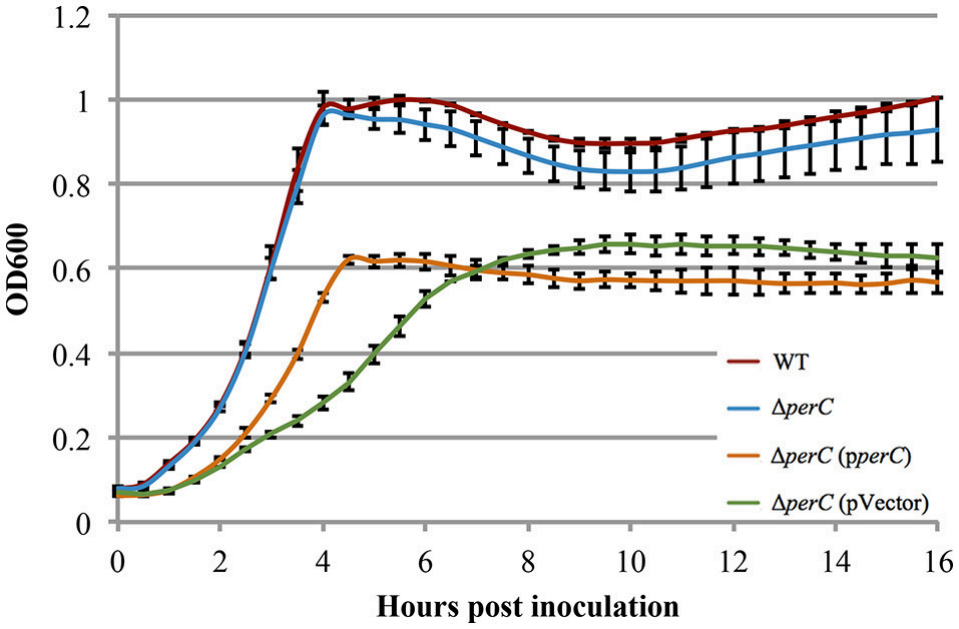
**Complement EPEC strain Δ***perC*** (p***perC***) and empty vector strain Δ***perC*** (pVector) have different growth rates when cultured in low-glucose DMEM (OD_**600**_ ± SE)**. Complement strain Δ*perC* (p*perC*) (orange) grows faster than Δ*perC* (pVector) (green) EPEC strains in low-glucose DMEM static culture with 15 μg/ml tetracycline during hours 2–4 in the exponential phase (*p* = 0.0004). WT EPEC strain E2348/69 (red) and coisogenic deletion strain Δ*perC* (blue) have similar growth rates in low-glucose DMEM static culture (without tetracycline) (*p* = 0.5748). Cultures were inoculated at the same concentration, and then the OD_600_ was measured every 30 min for 16 h. Exponential phase doubling times based on the linear fits for hours 2–4: WT (33 min), Δ*perC* (34 min), Δ*perC* (p*perC*) (63 min), and Δ*perC* (pVector) (81 min).

The WT EPEC and Δ*perC* mutant strains grew at similar rates shaking in LB (Figure S2). Likewise, the complemented Δ*perC* (p*perC*) and empty vector Δ*perC* (pVector) strains grew at similar rates under these conditions, albeit with greater doubling time compared to the WT and Δ*perC* strains, primarily due to the presence of antibiotic selection (Figure S2). The similar growth rates of the paired bacterial strains permitted us to make comparisons between these strains in subsequent assays. Low-glucose DMEM tissue culture medium mimics the intestinal environment more closely than LB, thus enhancing the expression of virulence proteins (Puente et al., 1996; Rosenshine et al., 1996; Leverton and Kaper, 2005). The plasmid-containing strains Δ*perC* (p*perC*) and Δ*perC* (pVector) grew at different rates between 2 and 4.5 h post-inoculation (*p* = 0.0004; Figure 2). The doubling time during this period of strain Δ*perC* (p*perC*) was 63 min compared to 81 min of strain Δ*perC* (pVector). The WT and Δ*perC* strains grew at similar rates in a static culture of DMEM (Figure 2). We concluded that overexpression of PerC conferred a growth advantage to the Δ*perC* (p*perC*) strain over the empty-vector strain Δ*perC* (pVector) during the exponential phase of growth when cultured in medium that mimics tissue culture conditions.

**Figure 3:**
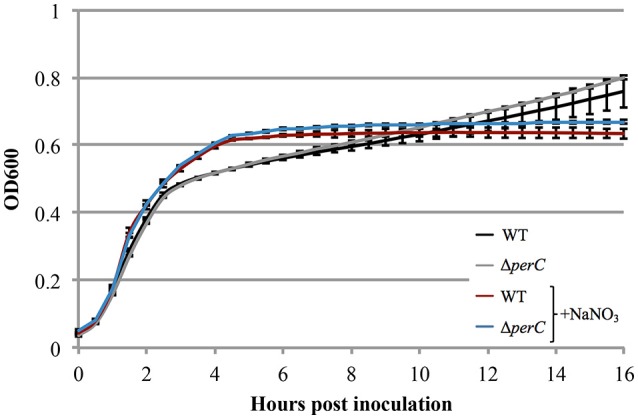
**WT EPEC strain E2348/69 and coisogenic deletion strain Δ***perC*** have similar growth rates when anaerobically cultured in tryptic soy broth with 0.5% (w/v) pig mucin, whether cultured with (+NaNO_**3**_) or without 40 mM sodium nitrate (NaNO_**3**_) (OD_**600**_ ± SE)**. Cultures were inoculated at the same concentration and the OD_600_ was measured every 30 min for 16 h. Linear regression of 0.5–4 h post-inoculation indicates that the growth rate of WT cultures were the same whether cultured with or without excess NaNO_3_ (*p* = 0.4489). Likewise, the growth rate of the Δ*perC* mutant was similar with and without NaNO_3_ (*p* = 0.4062). Exponential phase doubling times based on the pooled linear fits for hours 0.5–4: WT (34 min) and Δ*perC* (34 min).

**Figure 4:**
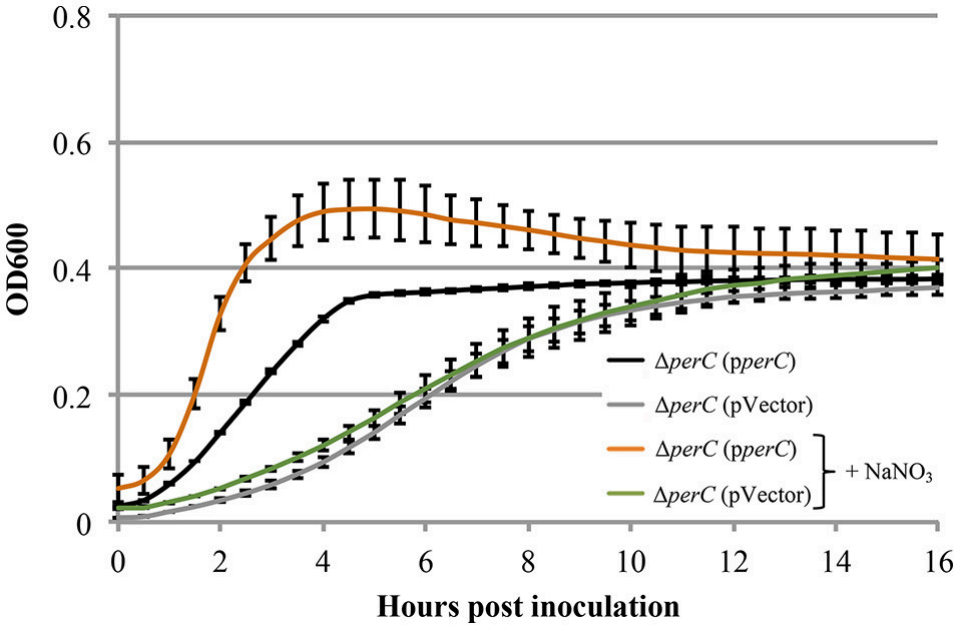
**EPEC strain Δ***perC*** (p***perC***) has a greater growth rate when anaerobically cultured in tryptic soy broth and 0.5% (w/v) pig mucin with added 40 mM sodium nitrate (+NaNO_**3**_) than without added NaNO_**3**_ (OD_**600**_ ± SE)**. Cultures were grown in the presence of 15 μg/ml tetracycline. Cultures were inoculated at the same concentration, and then the OD_600_ was measured every 30 min for 16 h. Linear regression of 0.5–4 h post-inoculation indicates that the growth rate of the Δ*perC* (p*perC*) cultures were greater in the presence of excess NaNO_3_ than in the absence (*p* = 0.0053). Linear regression revealed that the growth rate of the Δ*perC* (pVector) strain was similar with and without NaNO_3_ (*p* = 0.0896).

Based on the these data, we predicted that EPEC strains containing PerC would exhibit a growth advantage compared to strains not containing this regulator when grown in anaerobic conditions. We did not observe greater WT growth rate when grown in TSB with 0.5% mucin with oxyrase compared to that of the Δ*perC* mutant, or lower doubling time, in the presence of NaNO_3_ (Figure 3). However, the complemented strain Δ*perC* (p*perC*) showed significantly lower doubling time (70 min) than the empty vector strain Δ*perC* (pVector) (168 min) under anaerobic conditions induced by Oxyrase, and less still in anaerobic conditions with the addition of 40 mM NaNO_3_ (44 min) (Figure 4). Supported by the vector containing strains, these data collectively demonstrated that PerC overexpression conferred a growth advantage for EPEC bacteria respiring anaerobically in the presence of the terminal electron acceptor nitrate.

**Figure 5:**
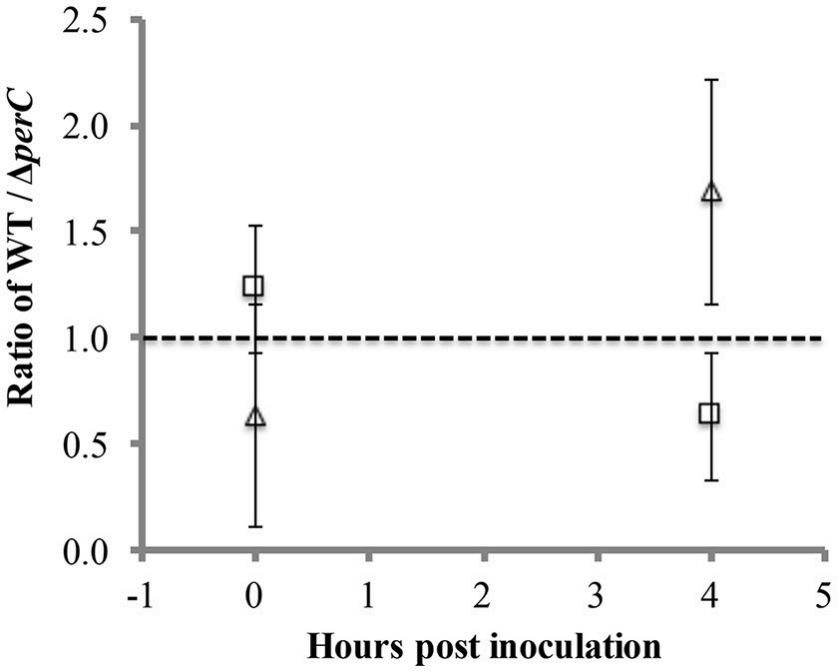
**Competition between WT and Δ***perC*** EPEC strains grown in low-glucose DMEM with (triangle) and without (square) 40 mM sodium nitrate**. Cultures inoculated with equal amounts of the WT and Δ*perC* strains were grown to mid-exponential phase, and relative abundance of each strain was determined by CFU/ml plate counts on LB and LB supplemented with 50 μg/ml kanamycin.

To provide a more sensitive measure of growth advantage, we conducted a competition assay between the WT and Δ*perC* strains in low-glucose DMEM with and without excess nitrate. We observed a difference just under significance of greater growth of the WT strain over the Δ*perC* strain in the presence of nitrate at 4 h post-inoculation (Figure 5). The ratio of WT to Δ*perC* growth in colony forming units per ml (CFU/ml) in DMEM with 40 mM sodium nitrate was nearly significantly greater than the growth ratio in DMEM after 4 h of growth (*p* = 0.0555; Figure 5). This ratio was not different between growth conditions at the point of inoculation (*p* = 0.3015). Interestingly, we observed that overexpression of PerC in the K-12, MC4100 laboratory strain of *E. coli* also conferred a growth advantage when grown anaerobically in TSB containing mucin and excess nitrate (Figure S3). Combined, these data indicated that PerC-containing EPEC, and perhaps many *E. coli* strains exhibit a growth advantage over strains lacking this regulator, particularly when overexpressed.

### PerC biases the *fim* switch to the OFF position

Our RNAseq data indicated less transcription of *fim* genes, encoding the type I pili, in the WT compared to the Δ*perC* deletion strain (Table 4). Likewise, the lesser transcription of *fimA* was confirmed by RT-qPCR in the PerC overexpressing strain compared to the empty vector control (Figure 1). As we observed 100-fold down-regulation of *fimA* by RNAseq, we suspected that this was not due to transcriptional control, but rather to phase variation mediated by the “*fim* switch” (*fimS*). A simple PCR-based assay, combined with a BstUI restriction enzyme digest is used to indicate the orientation of the *fimS* promoter element (Bower et al., 2005; Corcoran and Dorman, 2009; Schwan, 2011).

**Figure 6:**
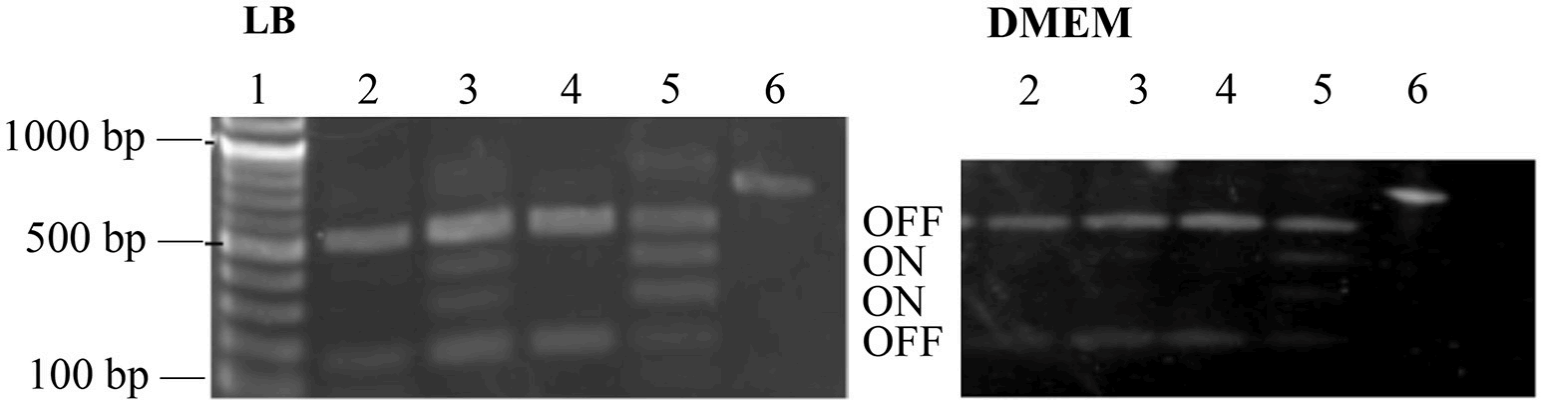
*****fimS*** region is biased to the OFF phase in strains with functional ***perC*****. BstUI digestion of amplified *fimS* gDNA reveals that *fimS* populations from EPEC strains with a functional *perC* gene, such as WT strain E2348/69 and complement strain Δ*perC* (p*perC*), primarily contain *fimS* DNA in the OFF orientation compared to strains with a mutated *perC* gene, such as coisogenic deletion strain Δ*perC* and Δ*perC* (pVector). Lanes (1) NEB 100 bp DNA ladder, (2) WT, (3) Δ*perC*, (4) Δ*perC* (p*perC*), (5) Δ*perC* (pVector), (6) unrestricted amplicon. Bacterial cultures were grown in LB (left) and DMEM (right).

Phase-ON bands were below the limit of detection for strains containing PerC, and *fimS* bands were primarily in the OFF orientation when grown both in LB and in low-glucose DMEM (Figure 6). The *fimS* assay showed phase-ON and phase-OFF bands, despite still having a pronounced OFF-bias, for the Δ*perC* and the Δ*perC* (pVector) empty control strains when strains were grown in both LB and DMEM. These assays provided evidence that PerC controls *fim* gene expression via the invertible phase variation mechanism.

## Discussion

In this report we defined the regulon of EPEC PerC, closely related to the PchABC proteins found in the EHEC pathotype, using RNA sequencing analysis. In support of our hypothesis that the plasmid-encoded PerC mediates niche adaptation, this regulator controls expression of genes involved in anaerobic respiration, notably nitrate and nitrite reduction (Table 3). We observed greater abundance of mRNA transcripts of *napC* and *narK* comparing the WT to the Δ*perC* mutant strain. The gene products from *nirB, nirC, and nrfA* detoxify nitrite by transport and reduction to ammonia, and the expression of these genes was also increased in the presence of PerC by our RNAseq analysis. These data support the role of PerC controlling anaerobic respiration using nitrate as a terminal electron acceptor. Consistent with these results, Hazen et al. observed increased expression of *napG* and *napH* using RNAseq for EPEC E2348/69 in early exponential phase when grown in DMEM (Hazen et al., 2015).

Measurable growth advantages were observed where PerC was expressed from a multi-copy plasmid (Figures 2, 4). When grown in the tissue culture medium DMEM (Figure 2) and anaerobically in TSB with pig mucin mimicking the intestinal niche, we observed a clear growth advantage for the strain expressing PerC from a plasmid vs. the empty vector control in the presence of nitrate (Figure 4). The growth advantage was maximal when PerC was over-expressed from the cloning vector when comparing growth in TSB plus mucin with and without added nitrate. In the WT strain, PerC is expressed from its native pEAF plasmid, present in slightly lower abundance (~2–5 copies) than the cloning vector pMPM-T3 (~10–12 copies) (Gibbs et al., 1993; Mayer, 1995). The growth advantage conferred to the complemented strain containing pTEPPerC1 compared to the empty vector control is likely demonstrable due to the slightly higher copy number of the pTEPPerC1 plasmid than the native pEAF plasmid in tEPEC. Additionally, the native promoter for the *perABC* operon drives the expression of PerC from the pEAF plasmid, while a *lac* promoter controls expression in pMTP-T3 (Mayer, 1995) (though IPTG is not necessary for induction). These data clearly indicate that PerC confers a growth advantage to EPEC in the presence of nitrate, consistent with our whole transcriptome and RT-qPCR analyses, and suggest the importance of gene dosage in PerC-mediated regulation. Further, when grown aerobically in LB, growth of the plasmid-containing strains is identical (Figure S2), so the observed phenotype is clearly dependent upon growth conditions. Lastly, our observations concerning gene dosage of *perC* in EPEC in the present study complement findings in EHEC showing that multiple copies of *pch* genes are important for T3SS expression (Porter et al., 2005).

While a control experiment clearly showed that *perC* expressed from a plasmid activates *LEE1* expression (Figure S1), we were surprised to not observe this regulation by RNAseq analysis when comparing the wt vs. *perC* deletion strain, and this might be explained, in part, by copy number. Another explanation might be that the tissue culture DMEM activates the LEE, in general, and thus there could be minor changes in expression under certain conditions when the LEE genes are already strongly expressed. With only one of many regulatory inputs, such as PerC, it would be difficult to see subtle changes in transcription. Another factor that could have minimized observed changes in transcriptional activity is that large operons, like those found in the LEE, are difficult to quantify in terms of regulation because, in the analysis, the number of reads of a particular mRNA is inversely proportional to the length in RNAseq. Thus, the power of the analysis is compromised by the operon organization of the LEE.

Concerning the plasmid-containing Δ*perC* strains constructed by mating, we acknowledge that conjugative helper plasmids can move IS or other genetic elements into recipients that might confound results. However, multiple biological and technical replicates bolster confidence in our data. Furthermore, the observation that the pTEPPerC1 plasmid conferred growth advantage to both pathogen and commensal strains (Figure S3), and that, approaching significance, the wt EPEC outcompeted the Δ*perC* strain when grown in conditions mimicking the human gut increased confidence that expression of PerC confers a measurable growth advantage to EPEC.

Illustrating the importance of anaerobic respiration in niche adaptation, *E. coli* deficient in nitrate or fumarate reductase activity have colonization defects in a mouse model of infection (Jones et al., 2011). We cannot rule out that the growth advantages we observed in the presence of PerC also could have been due, in part, to the presence of terminal electron acceptors other than nitrate in DMEM and/or TSB containing Oxyrase Broth (Figures 2, 4). DMEM contains ferric nitrate at 0.25 μM as a source of iron, and in the intestine, nitrate produced by the inflammatory response confers a growth advantage to commensal *E. coli* in mice (Winter et al., 2013). This inflammation produces nitrate levels similar to that found in DMEM (Lopez et al., 2015). Further, a *Salmonella napA* mutant defective in periplasmic nitrate reduction shows a growth defect in the lumen of the colon in a mouse model of infection (Lopez et al., 2015). Consistently, *Vibrio cholerae* and *Campylobacter jejuni* possess periplasmic nitrate reductases, while the cytosolic homologs are absent (Merrell et al., 2002; Sellars et al., 2002). These observations, combined with our results establish the role of PerC in EPEC niche adaption by controlling expression of the genes necessary for periplasmic nitrate reduction during anaerobic respiration.

PerC could also play a role in inducing nitrogen assimilatory pathways, which utilize *glnK* (2.1-fold change, *q* = 0.02) and *amtB* (1.4-fold change, n.s.) to sense intracellular glutamine and ammonia, respectively (van Heeswijk et al., 2013). Our lab's previous promoter trap assay identified *fnr* as a regulatory target of PerC (data not shown). Though we did not observe changes in transcript levels for *fnr* by RNAseq analysis (0.9-fold change, n.s.) many of the regulated metabolic genes identified were within its regulon (Table 3, Table S1), suggesting the possibility that FNR, a master switch between aerobic and anaerobic metabolism in *E. coli*, might play a role in this the observed regulation, perhaps post-transcriptionally (Constantinidou et al., 2006).

Further, the observation that WT EPEC shows increased *rhyB* expression compared to the Δ*perC* strain correlates with the induction of nitrite reductases (Table S1). Homologs of *ryhB* in *S. enterica* were recently shown to induce expression of the *nirBD* operon and mediate cellular resistance against nitrosating agents (Calderon et al., 2014). This provides a connection between PerC-induced regulation of nitrite reductases and non-coding RNAs, and suggests that PerC accomplishes some portion of its regulation of metabolic genes though Hfq-dependent sRNAs.

The manipulation of surface antigens is also an important component of niche adaptation leading to pathogenesis. We present evidence that PerC biases the *fimS* promoter element into the OFF orientation, decreasing expression of the type I fimbriae. RNAseq data, RT-qPCR, and the genomic *fimS* assay support this conclusion (Table 4, Figures 1, 6). Depending on environmental conditions, the invertible promoter element *fimS* can be inverted by two site-specific recombinases, encoded by the genes *fimB* and *fimE*. These two recombinases act independently of each other on *fimS*. FimB inverts *fimS* to the ON and OFF orientations equally, whereas FimE rapidly converts the *fimS* orientation from ON to OFF (Corcoran and Dorman, 2009). Microarray analysis elucidating the Ler regulon showed repression of the *fim* operon (Bingle et al., 2014), and with PerC known to regulate *LEE1* (Mellies et al., 1999; Bustamante et al., 2011) our results suggest that PerC diminishes *fim* expression indirectly through Ler. The observed ~100-fold *fim* down-regulation is achieved through phase variation, with Ler most likely interacting with regulatory elements controlling expression of the FimE recombinase that biases the switch to the OFF position (Corcoran and Dorman, 2009). This regulation, the strongest we observed for PerC, most likely has innate immune implications because the FimH tip adhesin of the type I fimbriae induces IL-8 and TNFα expression through binding to TLR4 and inducing MyD88 signaling (Mossman et al., 2008).

In uropathogenic *E. coli* (UPEC), the type I fimbriae enhance urinary tract infections (Connell et al., 1996; Martinez et al., 2000). During ascending infections, the *fim* switch changes to the OFF orientation in UPEC, so that the *pap* pili can be the dominant surface antigen (Lane and Mobley, 2007). The pyelonephritis-associated pili are associated with sudden and severe kidney infections (Jantunen et al., 2000; Tseng et al., 2001). In EPEC, our data suggests feedback regulation of the *bfp* operon through the PerA regulator (Table S1). Thus, because PerC activates expression of the type III secretion system in EPEC through Ler (Mellies et al., 1999; Bustamante et al., 2001, 2011; Porter et al., 2005), we propose that PerC functions to preference expression of the type III secretion apparatus to enhance attaching and effacing lesion formation at the site of EPEC attachment, the distal small intestine, establishing a local infection.

tEPEC containing *perABC* exhibit a localized adherence pattern on intestinal epithelial cells mediated by the BFP and can form type III secretion system-dependent attaching and effacing (A/E) lesions within 3 h post-infection, while aEPEC lacking the pEAF plasmid require ~6 h to form A/E lesions (Bueris et al., 2015). Placing the *per* locus into aEPEC shortens the timeline whereby A/E lesions form in the 3-h time period, in part, by PerC-regulation of the LEE. Clinically, tEPEC cause acute disease, whereas aEPEC strains cause a more chronic, persistent diarrhea (Afset et al., 2004; Nguyen et al., 2006). Furthermore, the isolation of aEPEC from asymptomatic individuals (Ochoa et al., 2008) has led some investigators to question whether these isolates are truly pathogens, or simply innocent bystanders (Hu and Torres, 2015). Our results are consistent with these observations. PerC of the tEPEC strain E2348/69 confers a growth advantage when grown anaerobically in the presence of nitrate, and promotes preferential display of the type III secretion system. Thus, PerC contributes to acute disease caused by tEPEC, and we suspect this also to occur in EHEC strains containing the PchABC homologs, by similar or related mechanisms.

## Author contributions

Experiments were performed by the following authors: Preparation for and analysis of RNA sequencing data–JO; growth curves, competition assays, and qPCR–AP; *fimS* assays–LB. The manuscript was written by JLM and AP, and reviewed by all authors before submission. Thanks also to Gus Kilgore for critical review of the manuscript.

## Funding

This work was supported by NIH grant 1R21AI115193-01 awarded to JLM and Reed College Biology Undergraduate Research Awards to JO and LB.

### Conflict of interest statement

The authors declare that the research was conducted in the absence of any commercial or financial relationships that could be construed as a potential conflict of interest.

## Footnotes

^1^Sas Institute Inc JMP version 11. Cary, NC, 1989-2007.

^2^Graphpad Software Prism version 7, La Jolla CA.

## Supplementary material

Table S1 **RNA-sequencing data of genes significantly regulated by PerC in EPEC**. The fold change of fragments per kilobase mapped (FPKM) was determined by comparing transcriptome expression between WT EPEC and the coisogenic Δ*perC* mutant. Significance was determined using a false-discovery rate (FDR) of 10% (*q* ≤ 0.10).

Figure S1 **Plasmid pTEPPerC1 contains a functional ***perC*** gene that activates transcription of the ***LEE1*** operon**. Strain FL4101Φ*LEE1-lacZ* was made electrocompotent and transformed with the plasmid containing a functional *perC* gene (pTEPPerC1) or the empty vector (pMPM-T3). β-galatctosidase activity (in Miller units) in the transformant FL4101 (pTEPPerC1) was greater than that of the transformant FL4101 (pMPM-T3) (*p* < 0.0001). The positive control MC4100 (pJLM164) with a multi-copy *lacZ* gene under its native promoter generated a relatively high amount of activity, and the negative control FL4101 not transformed with a plasmid generated minimal activity.

Figure S2 **Growth rates of experimental bacterial strains not different when cultured shaking in LB over 14 h (OD_**600**_ ± SE)**. WT EPEC strain E2348/69 (red) and coisogenic deletion strain Δ*perC* (blue) have similar growth rates when grown in LB, as do Δ*perC* (p*perC*) (orange) and Δ*perC* (pVector) (green) EPEC strains. The latter strains were grown with the addition of 15 μg/ml tetracycline.

Figure S3 **PerC confers a growth advantage to lab strain MC4100 (OD_**600**_ ± SE)**. Strains MC4100 (p*perC*) and MC4100 (pVector) grown in tryptic soy broth containing 0.5% mucin, made anaerobic by Oxyrase for Broth, with 15 μg/ml tetracycline, and with and without added 40 mM sodium nitrate (NaNO_3_). By linear regression, strain MC4100 (p*perC*) has a greater growth rate during the exponential phase (hours 2–4) when grown in the presence of excess nitrate than solely under anaerobic conditions (*p* < 0.0001). MC4100 (p*perC*) has a greater growth rate than MC4100 (pVector) in the exponential phase when grown in anaerobic conditions at hours 2–4 (<0.0001). There is no difference in growth between strains MC4100 (p*perC*) and MC4100 (pVector) when statically grown in LB (data not shown).
