# Supplementary figures and images for: PerC Manipulates Metabolism and Surface Antigens in Enteropathogenic Escherichia coli
Source: Front Cell Infect Microbiol. 2017 Feb 7;7:32. doi: 10.3389/fcimb.2017.00032 (PMC5293775; doi:10.3389/fcimb.2017.00032)

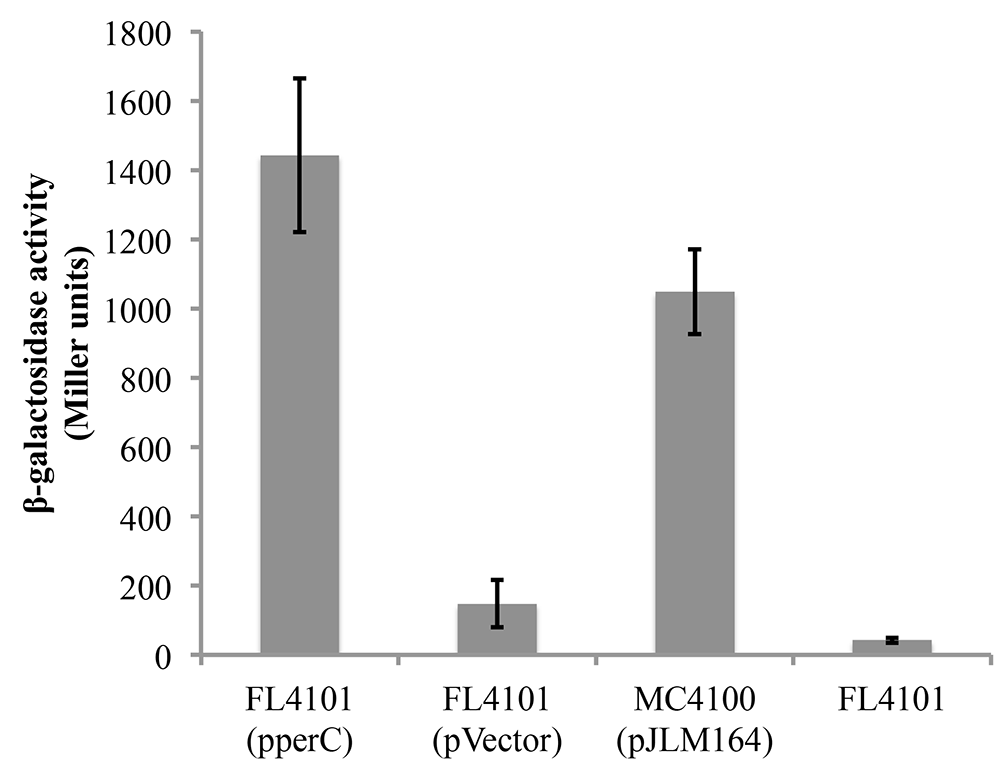

Supplement: Figure S1 — Plasmid pTEPPerC1 contains a functional perC gene that activates transcription of the LEE1 operon. Strain FL4101ΦLEE1-lacZ was made electrocompotent and transformed with the plasmid containing a functional perC gene (pTEPPerC1) or the empty vector (pMPM-T3). β-galatctosidase activity (in Miller units) in the transformant FL4101 (pTEPPerC1) was greater than that of the transformant FL4101 (pMPM-T3) (p < 0.0001). The positive control MC4100 (pJLM164) with a multi-copy lacZ gene under its native promoter generated a relatively high amount of activity, and the negative control FL4101 not transformed with a plasmid generated minimal activity. [file Image1.TIF]

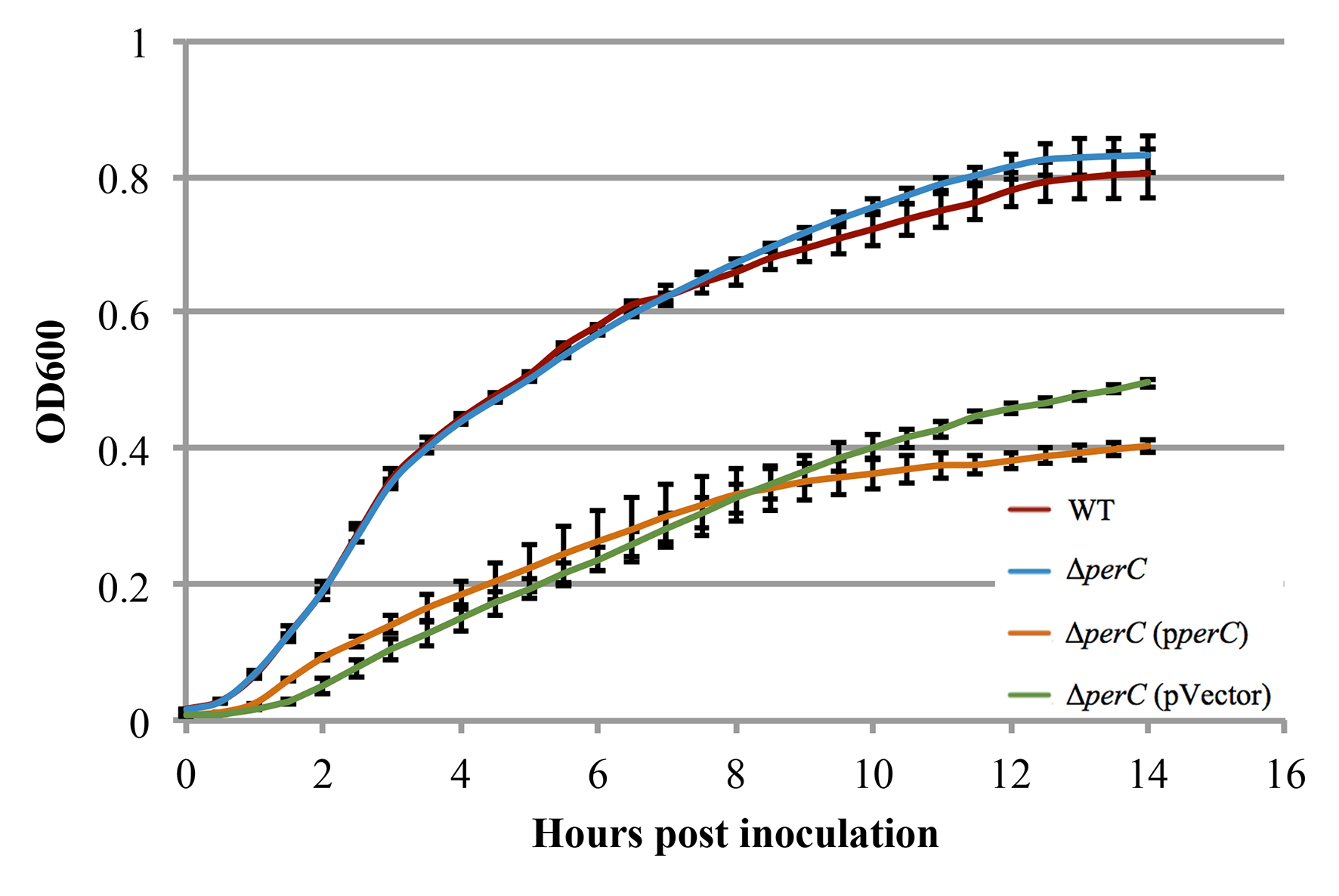

Supplement: Figure S2 — Growth rates of experimental bacterial strains not different when cultured shaking in LB over 14 h (OD600 ± SE). WT EPEC strain E2348/69 (red) and coisogenic deletion strain ΔperC (blue) have similar growth rates when grown in LB, as do ΔperC (pperC) (orange) and ΔperC (pVector) (green) EPEC strains. The latter strains were grown with the addition of 15 μg/ml tetracycline. [file Image2.TIF]

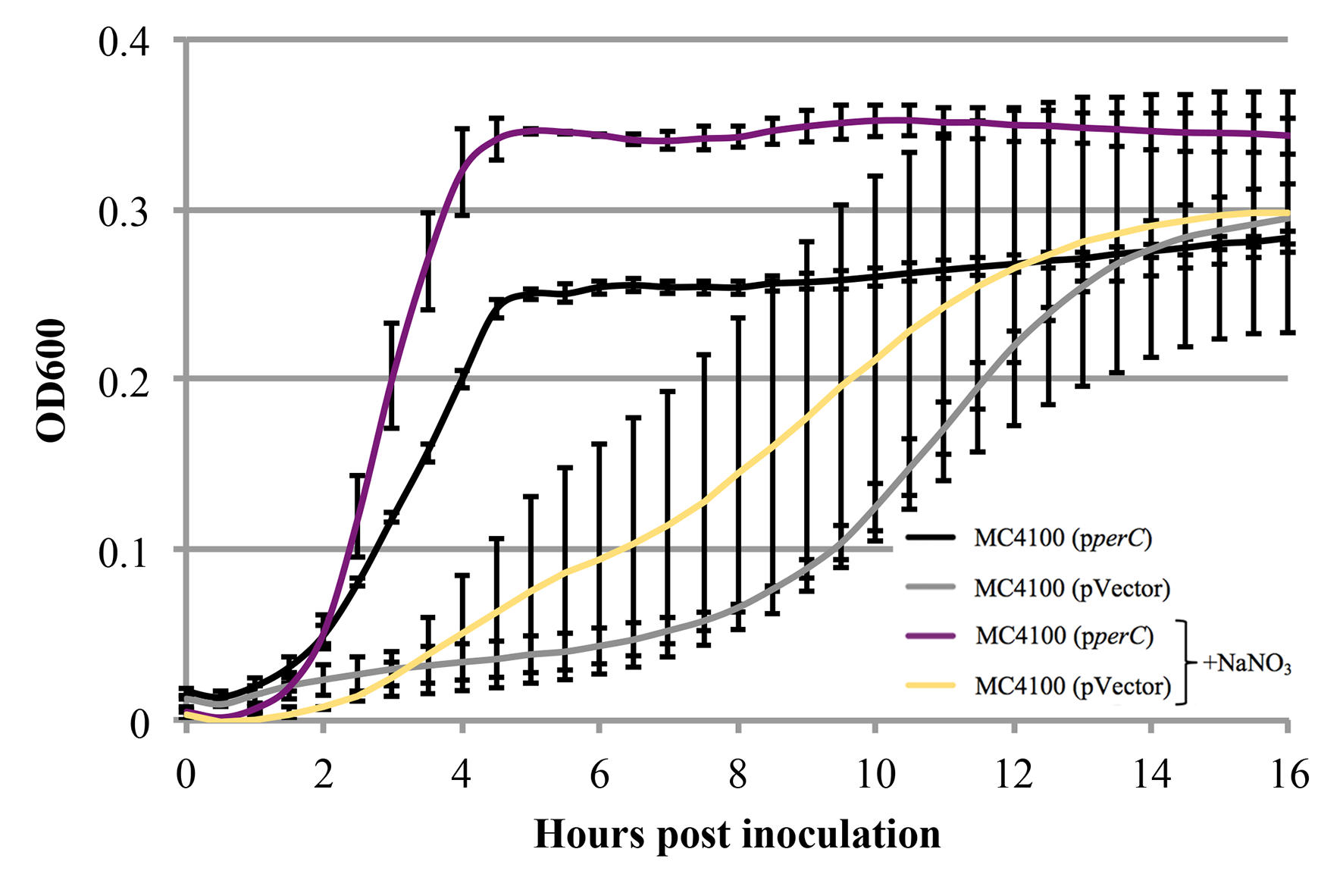

Supplement: Figure S3 — PerC confers a growth advantage to lab strain MC4100 (OD600 ± SE). Strains MC4100 (pperC) and MC4100 (pVector) grown in tryptic soy broth containing 0.5% mucin, made anaerobic by Oxyrase for Broth, with 15 μg/ml tetracycline, and with and without added 40 mM sodium nitrate (NaNO3). By linear regression, strain MC4100 (pperC) has a greater growth rate during the exponential phase (hours 2–4) when grown in the presence of excess nitrate than solely under anaerobic conditions (p < 0.0001). MC4100 (pperC) has a greater growth rate than MC4100 (pVector) in the exponential phase when grown in anaerobic conditions at hours 2–4 (<0.0001). There is no difference in growth between strains MC4100 (pperC) and MC4100 (pVector) when statically grown in LB (data not shown). [file Image3.TIF]
